# Endocrine and metabolic late effects following cancer treatment: challenges and controversies

**DOI:** 10.1530/EC-22-0261

**Published:** 2022-06-09

**Authors:** Judith Gebauer, Claire E Higham

**Affiliations:** 1 Department of Internal Medicine I, University Hospital Schleswig-Holstein, Luebeck, Germany; 2 Department of Endocrinology, Christie Hospital NHS Foundation Trust, University of Manchester, and Manchester Academic Health Science Centre, Manchester, UK

In 2020, there were 19.3 million new cancer cases diagnosed globally (1, 2), and numbers continue to rise. During the last few decades, cancer survival has improved considerably; in the 1960s, children had a 5-year cancer survival rate of around 25% which has risen to over 80% in high-income countries (3, 4). Rising incidence and improved survival rates have resulted in a growing number of long-term cancer survivors; currently over 43 million worldwide (5, 6).

Although cured of their cancer, these patients continue to present a challenge to health systems as the majority will develop sequelae from their cancer or cancer treatments occurring years to decades after the end of cancer treatment (Fig. 1). These late consequences are reflected in a range of different organs and functions with varying severity, from mild restrictions to new life-threatening diseases and can have significant effects on the quality and quantity of life, thus requiring a multidisciplinary approach to care (7). Up to half of them will develop endocrine or metabolic consequences.

**Figure 1:**
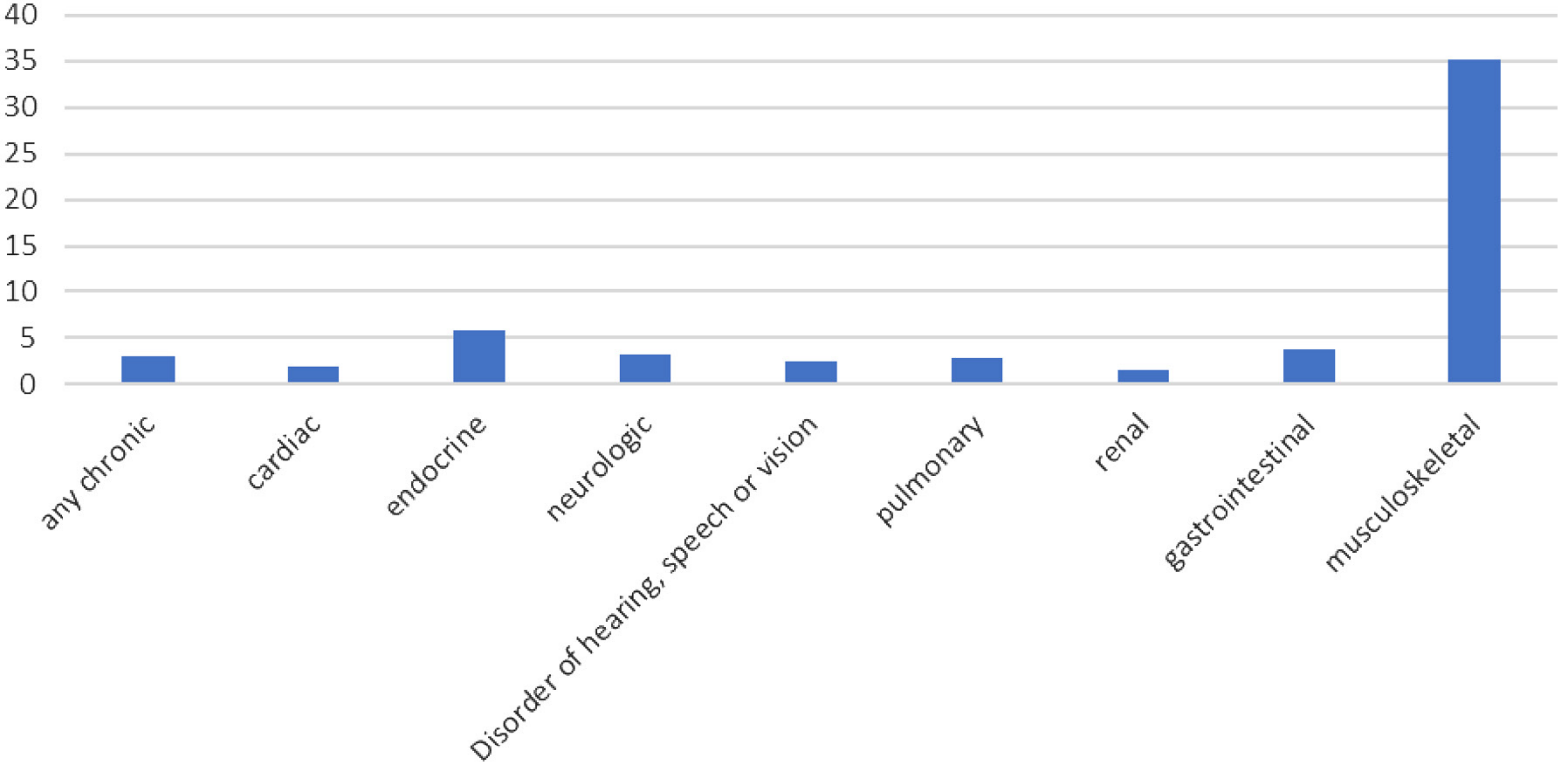
Relative risks for chronic health conditions in childhood cancer survivors (compared to their siblings) (9).

Childhood cancer survivors represent a comparably small group of patients in this large cohort that have been studied for decades due to their excellent long-term survival rates and the long life span ahead of them after the end of treatment. Much knowledge about late effects is therefore derived from this patient group and serves as the basis for guidelines and recommendations, for example, the International Guideline Harmonisation Group (https://www.ighg.org/) who promote risk-adapted long-term follow-up with the aim of facilitating early detection and treatment of possible sequelae (8). With the focus shifting from survival to living beyond cancer in many, optimal long-term follow-up is essential to reduce long-term morbidity and mortality as well as to ensure good quality of life in all long-term cancer survivors.

Developing evidence-based research and guidance for late effects is challenging for a number of reasons, including rapidly evolving oncology treatments, individualized cancer regimens and the duration of time between cancer treatments, and the development of sequelae. This has been compounded by relatively few studies investigating the underlying pathophysiology of late effects and lower levels of research funding compared to other areas of oncology research. These challenges need to be addressed as cancer incidence and cancer survival rates rise.

Despite increasing awareness of late effects, implementation of long-term follow-up care still varies considerably between nations and patient cohorts. Specialized late effects centers have been established in some countries during the last decade, offering standardized care for a few; however, large numbers of patients receive only basic follow-up care or, after the end of oncological follow-up care, no long-term follow-up care at all. Therefore, it is of enormous importance to address this inequity and determine which examinations at which frequency should be performed to detect possible long-term health consequences in a timely fashion, without overburdening the resources of health care systems and without exposing patients to unnecessary risks that may arise from overdiagnosis.

Articles in the series will highlight other current controversies in the field of late effects of cancer in order to present pragmatic approaches to optimal care. Moreover, expert authors from different nations and specialties will explore their topic from a range of angles, representing the multidisciplinary approach as well as the need for continuous international collaboration between specialists. Topics will cover bone health, growth hormone deficiency, the impact of different radiotherapy techniques on the hypothalamic–pituitary axis, risk assessment for second cancers, as well as strategies for international collaboration and different approaches to data analysis.

This special collection is designed to initiate discussions and critique of currently available recommendations, to evaluate current knowledge, to highlight the importance of the continuous adaption and development of clinical and mechanistic research around late effects, and to emphasize the value of surveillance strategies to ensure optimal long-term follow-up for every cancer survivor. To accompany the invited short review articles, we welcome the submission of original research articles in the field of late effects to complement and enhance this special collection.

## Declaration of interest

The authors declare that there is no conflict of interest that could be perceived as prejudicing the impartiality of this editorial.

## Funding

This work did not receive any specific grant from any funding agency in the public, commercial, or not-for-profit sector.
